# Palmitoylation as a Key Regulator of Ras Localization and Function

**DOI:** 10.3389/fmolb.2021.659861

**Published:** 2021-03-17

**Authors:** Carla Busquets-Hernández, Gemma Triola

**Affiliations:** Department of Biological Chemistry, Laboratory of Chemical Biology, Institute of Advanced Chemistry of Catalonia (IQAC-CSIC), Barcelona, Spain

**Keywords:** Ras, palmitoylation, acylation, lipid posttranslational modifications, cancer, membrane subdomains, thioesterases

## Abstract

Ras proteins require membrane association for proper function. This process is tightly regulated by reversible palmitoylation that controls not only the distribution over different subcellular compartments but also Ras compartmentalization within membrane subdomains. As a result, there is a growing interest in protein palmitoylation and the enzymes that control this process. In this minireview, we discuss how palmitoylation affects the localization and function of Ras proteins. A better understanding of the regulatory mechanism controlling protein lipidation is expected to provide new insights into the functional role of these modifications and may ultimately lead to the development of novel therapeutic approaches.

## Introduction

**FIGURE 1:**
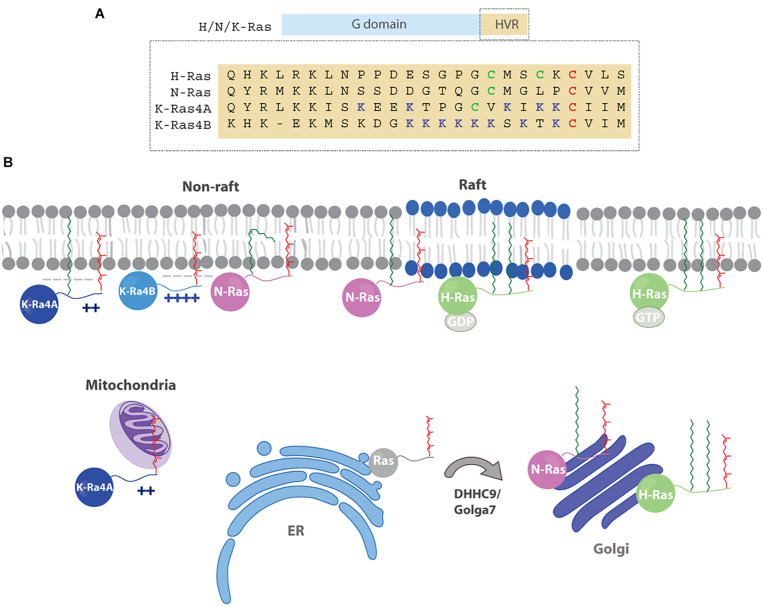
**(A)** The Ras isoforms contain a highly homologous G domain (90%) and a C-terminal hypervariable region (HVR) that comprises the last 24 amino acids. The HVR exhibit a low degree of homology between isoforms (∼ 10%) and present different post-translational lipid modifications. Red cysteines (C) are farnesylated, green cysteines (C) are palmitoylated, blue lysines (K) are polybasic residues. **(B)** Ras membrane distribution is dynamic and depends on membrane targeting motifs (polybasic sequences and lipids) and changes in palmitoylation state that combined, modulate Ras trafficking and localization to specific membranes (PM, endomembranes, subdomains). Hence, farnesylated H/N-Ras get palmitoylated at the Golgi apparatus by DHHC9/Golga7 and then are transferred to the PM via the secretory pathway. After depalmitoylation, H/N-Ras traffic back to the Golgi to be reacylated. Due to the presence of two palmitoyl moieties H-Ras gets enriched in the PM, whereas N-Ras is predominantly localized at the Golgi. Once in the membrane, H-Ras segregates in different microdomains (rafts, non-rafts) in a GDP/GTP dependent manner. Palmitoylated N-Ras associates preferentially with raft/non-raft boundary regions, although a N-Ras protein modified with an unsaturated palmitoleic shows preferential accumulation in fluid domains. The localization of K-Ras4A on the PM relies on the presence of polybasic regions and a palmitoylated cystine, whereas K-Ras4B is palmitoylation independent. After depalmitoylation, K-Ras4A travels to the mitochondria and binds HK1.

The Ras superfamily of small GTPases comprises more than 150 monomeric G proteins. Their ability to act as molecular switches upon stimulation by upstream signals, alternating between the GTP-bound active state and the inactive GDP-bound form, allows Ras proteins to play a role in a diverse array of biological processes such as cell proliferation, signaling, differentiation and survival (Simanshu et al., 2017). Some of the most prominent members of the Ras superfamily are the four Ras isoforms which are encoded by three different genes: H-Ras, N-Ras and K-Ras that generates two splice variants, K-Ras4A and K-Ras4B. The four isoforms share a highly conserved G domain but mainly differ in the hypervariable region (HVR) which comprises the last 24 amino acids and several posttranslational modifications. Hence, all proteins undergo a three-step maturation pathway at the C-terminus known as CaaX box processing (Lowy and Willumsen, 1989; Ahearn et al., 2012) which includes farnesylation of the cysteine, proteolytic cleavage of the last three amino acids (aaX) (Boyartchuk et al., 1997) and carboxyl methylation (Clarke et al., 1988; Dai et al., 1998). Since the prenyl moiety is essential but not sufficient to mediate the stable membrane association required for proper signaling, all isoforms display additional membrane targeting motifs (Hancock et al., 1990). N-Ras and H-Ras are both palmitoylated at either one or two cysteine residues, respectively. K-Ras4B contains a polybasic stretch of eight lysines and K-Ras4A presents a palmitoylated cysteine and two polybasic regions (Figure 1A). As a result of these differences, the four isoforms show distinct subcellular localization and distribution in membrane microdomains, and generate distinct signaling outputs (Rocks et al., 2005). However, other factors can also influence Ras signaling. Thus, apart from the HVR, the G-domain and its modifications (ubiquitination, sumoylation, acetylation, glucosylation and nitrosylation) may also contribute to a particular membrane orientation and isoform specific signaling (Kapoor et al., 2012; Ahearn et al., 2018). In addition, some functional redundancy has been suggested for the different isoforms, as although only K-Ras is essential for normal mouse embryogenesis, its function can be replaced by H-Ras, however, associated to significant cardiotoxicity (Potenza et al., 2005).

Ras proteins are among the most frequently altered oncogenes in human cancers (Hobbs et al., 2016) and overall, approximately 20% of cancer patients harbor Ras mutations (Prior et al., 2020). Point mutations occur in hotspots codons (mainly 12, 13, and 61) and lead to constitutively active proteins resulting in uncontrolled proliferation. However, the prevalence of each isoform in human cancers is not uniform. K-Ras is by far the most frequently mutated isoform (76%), whereas N-Ras contributes to the 17% of human cancers and H-Ras to the remaining 7%. Furthermore, each isoform is related to different types of cancer. While K-Ras is usually associated to lung, colorectal and pancreatic cancers, N-Ras is more predominant in skin melanomas and H-Ras in bladder carcinomas (Prior et al., 2012).

All the above mentioned factors reveal the increasing complexity of Ras biology. From one side, Ras signaling capacity and functional heterogeneity depends on specific isoforms and mutations. However, the extent to which the lipidation state determines the resident time, the specific subcellular localization or the partition into different membrane subdomains, and by doing so, it enables accessibility to a preferential set of effector proteins, is poorly understood. In this minireview we will discuss how changes in the acylation pattern influence the spatial and functional heterogeneity of Ras proteins.

## Subcellular Localization and Function

### H-Ras and N-Ras

The localization of palmitoylated isoforms is determined by the reversible nature of this modification. Although S-acyl groups of some proteins do not turn over or they do it at a very low rate, some other proteins, such as the Ras isoforms, show a very rapid cycling. Thus, after palmitoylation at the Golgi by palmitoyl acyl transferases (PATs) (Stix et al., 2020), N/H-Ras are transferred to the plasma membrane (PM) via the secretory pathway. In their way to the PM, fully palmitoylated and active H-/N-Ras can also localize at recycling endosomes (Misaki et al., 2010). Next, depalmitoylation is mediated by thioesterases (Won et al., 2018) and occurs everywhere in the cell. Depalmitoylated Ras then traffics back to the Golgi where it can be reacylated (Rocks et al., 2005, 2010; Figure 1B). Due to the presence of two fatty acid moieties, depalmitoylation of H-Ras takes longer causing enrichment at the PM, whereas N-Ras, bearing only one palmitate, is predominantly localized at the Golgi. Moreover, the combined action of PATs and thioesterases results in an acylation/deacylation cycle that has a shorter half-life than that of the protein (∼6 min for N-Ras and around 20 min for H-Ras vs. ∼24 h protein half-life) (Magee et al., 1987) and introduces an additional level of regulation in the spatial and temporal modulation of Ras signaling (Rocks et al., 2005, 2010). Interestingly, marked differences can exist in the turnover rates of oncogenic and wild type Ras, despite sharing similar subcellular localizations (Baker et al., 2003).

#### H-Ras

H-Ras gets palmitoylated by DHHC9/Golga7, a member of the Asp-His-His-Cys (DHHC) family of PATs that comprises 23 different proteins. Additional involvement of DHHC18 has also been suggested (Swarthout et al., 2005; Yokoi et al., 2016). Thioester cleavage was initially proven by APT1 (Duncan and Gilman, 1998), APT2 (Tomatis et al., 2010) and the lysosomal PPT1 (Camp and Hofmann, 1993; Verkruyse and Hofmann, 1996). The interaction of H-Ras with both APT1/2, mainly occurring at the PM, was also confirmed by FRET studies (Pedro et al., 2017). More recently, the involvement of other thioesterases has also been suggested since the disruption of APT1 gene in yeast did not completely abolished H-Ras deacylation (Duncan and Gilman, 2002). ABHD17, a member of the mammalian α,β hydrolase-domain (ABHD) family of serine hydrolases (SH) has been shown to deacylate an overexpressed H-Ras in HEK293T cells, but this effect could not be observed in neurons (Yokoi et al., 2016). As the SH family consists of over 100 members and most of them have not known substrate yet, it can not be discarded that additional thioesterases acting on H-Ras may be identified in the future.

Apart from the enzymes involved in de/acylation, FKBP12 may add an additional layer of regulation by controlling the time of residence of H-Ras at the PM. FKBP12 promotes the *cis/trans* isomerization of the peptidyl-prolyl bond at position 178–179, which facilitates depalmitoylation probably by rendering the thioester bond accessible to membrane associated thioesterases. Interaction of FKBP12 with N-Ras has also been detected, but not with K-Ras (Ahearn et al., 2011).

Some studies have suggested that the individual palmitoyl residues may have different roles. Thus, whereas a C184S mutant was present at both the PM and the Golgi, a C181S mutant was mostly localized at Golgi (Roy et al., 2005a). Moreover, the deacylation rate of the C184S mutant significantly increased upon overexpression of APT2, whereas the rate of the C181S mutant did not change (Pedro et al., 2017). Studies with monopalmitoylated mutants may shed light on the role and substrate specificity of these positions. However, results should be interpreted with caution since singly palmitoylated H-Ras species do not seem to be present in cells (Yokoi et al., 2016).

Because of the continuous cycle of de/acylation, H-Ras populations are present at and signal from both the PM and the Golgi apparatus under steady-state conditions. However, functional Ras can also signal from additional subcellular compartments, such as the Endoplasmic Reticulum (ER) (Chiu et al., 2002; Fehrenbacher et al., 2009) and the differential subcellular localizations contribute to its wide signaling repertoire. Thus, organelle-specific interaction with effectors may be behind the variety of biological responses observed, such as proliferation (Chiu et al., 2002; Arozarena et al., 2004) or apoptosis (Herrero et al., 2016; Casar et al., 2018). Studies with engineered proteins have provided insight into H-Ras biology and its relationship with effector proteins. Hence, an active H-Ras directed to Golgi or ER led to the correlation of signaling outputs with defined subcellular protein pools (Matallanas et al., 2006; Agudo-Ibáñez et al., 2007) and enabled the identification of organelle-specific protein-protein interactions (Santra et al., 2019). Specific interactions were also unveiled employing an engineered exchange factor able to activate different subcellular pools of endogenous H-Ras (Herrero et al., 2020). In addition, activation at distinct subcellular sites also provides a temporal control of signaling, that is transient and rapid at the PM but slower and sustained at Golgi.

#### N-Ras

The singly palmitoylated **N-Ras** is predominantly localized at the Golgi apparatus under steady-state conditions. Palmitoylation of N-Ras is also mediated by DHHC9/Golga7 and, similarly to H-Ras, N-Ras can be depalmitoylated by the broad substrate-tolerant APT1 and APT2 (Rocks et al., 2010; Görmer et al., 2012; Vartak et al., 2014). Depalmitoylated N-Ras is then transported to the Golgi by the chaperone PDE6δ (phosphodiesterase of retinal rod subunit δ) which binds the prenyl group and enhances the cytoplasmic diffusion of the protein. PDE6δ can also transport K-Ras4B (Chen et al., 2010) and facilitates its delivery to membranes (Weise et al., 2012), but has much less effect on H-Ras. The reason behind this selectivity may be the degree of palmitoylation that negatively affects binding with PDE6δ, as only 25% of H-Ras is depalmitoylated at steady-state compared to the 50% of N-Ras (Chandra et al., 2012; Zimmermann et al., 2013). Depletion of PDE6δ or small-molecule based inhibition of Ras-PDE6δ interaction results in Ras mislocalization and consequently, in attenuated signaling (Chandra et al., 2012; Zimmermann et al., 2013).

Because lipidation impairment causes Ras mislocalization, significant efforts have been made to identify thioesterase inhibitors that might be not only interesting for fundamental research but also offer potential applications in drug discovery (Table 1). As a result, small-molecule inhibitors of thioesterases have emerged as key players in the study of de/acylation processes. The first potent APT1/2 inhibitors were the β-lactones Palmostatin B and M that led to impaired localization and signaling of N-Ras and H-Ras (Dekker et al., 2010; Hedberg et al., 2011; Rusch et al., 2011). However, the role of APT1/2 was later questioned, since their overexpression showed little effect on N-Ras localization (Agudo-Ibáñez et al., 2015) and selective inhibitors of APT1 or APT2 could not preserve the palmitoylation state of N-Ras (Adibekian et al., 2010a,b). Currently, there are accumulating evidences indicating that other thioesterases might contribute to the regulation of N-Ras palmitate turnover. Relevant candidates are the three isoforms ABHD17A/B/C, localized to PM and Rab6- and Rab11-positive endosomes and also targeted by Palmostatin M (Lin and Conibear, 2015). Thus, overexpression of ABHD17 redistributed N-Ras from the PM to intracellular membranes and a selective inhibitor of ABHD17, ABD957, has shown to inhibit the growth of cells that depend on N-Ras as an oncogenic driver (Remsberg et al., 2020). However, since ABD957 only partially impairs N-Ras depalmitoylation, additional, yet unknown, thioesterases may not be discarded (Lin and Conibear, 2015). Apart from the *cis/trans* isomerase FKBP12 mentioned above, an additional chaperone protein, VPS35, has also been involved in the regulation of N-Ras subcellular trafficking (Zhou et al., 2016).

**TABLE 1:** Enzymes and proteins that have been implicated in Ras metabolism and trafficking.

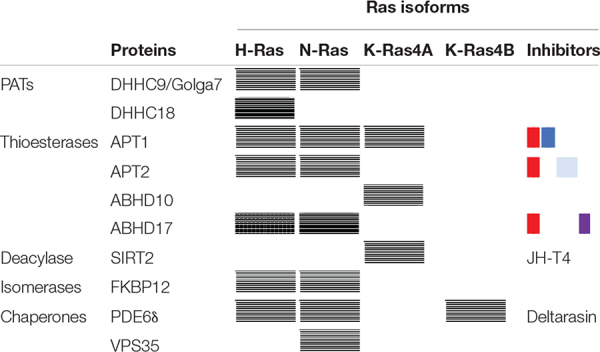

*Known inhibitors targeting these proteins are depicted on the right columns. Selectivity profile of thioesterase inhibitors: red, Palm M; dark blue, ML348, light blue, ML349; purple, ABD957.*

#### K-Ras

The K-Ras gene has two splice variants, K-Ras4A and K-Ras4B, both of them encoding oncogenic proteins when K-Ras is activated by mutation. It has been long considered that K-Ras4A was the minor splice variant and that its contribution to oncogenesis or tumor maintenance was negligible. However, RT-qPCR-based measurements revealed that K-Ras4A accounted for 10–50% of total K-Ras in cell lines derived from colon carcinoma and melanoma, and the relative abundance of K-Ras4A was even higher in primary human tumors (Tsai et al., 2015). All together, these recent advances have renewed the interest in the K-Ras4A isoform as a potential therapeutic target.

The two K-Ras splice variants have distinct mechanism of subcellular trafficking. Both variants require an essential farnesyl moiety, but localization and trafficking of K-Ras4B relies on the presence of polybasic residues that anchor the protein to the inner leaflet of the PM, whereas the membrane-targeting signals in K-Ras4A are two polybasic regions and an additional palmitoyl group, that independently contribute to the PM localization and signal output. Hence, only mutation of either region combined with loss of palmitoylation caused a significant reduction in ERK phosphorylation (Tsai et al., 2015) or abolished the ability to induce leukemia in mice (Zhao et al., 2015). Interestingly, in contrast to K-Ras4B and N-Ras, PDE6δ does not seem to function as a cytosolic chaperone for K-Ras4A (Tsai et al., 2015). K-Ras has also been implicated in the biogenesis of exosomes, tiny extracellular vesicles involved in cell-cell communication that have been also considered potential Ras signaling pathways (Sexton et al., 2019).

Recently, super-resolution immunofluorescence microscopy studies confirmed that the non-palmitoylated form of K-Ras4A also localizes on the outer mitochondrial membrane, where it specifically interacts with Hexokinase 1 (HK1), an enzyme that initiates glucose metabolism. Upon binding, K-Ras4A blocks the allosteric inhibition of HK1 resulting in an enhanced glucose consumption, which might contribute to the metabolic reprogramming of tumor cells aimed to sustain rapid tumor growth (Amendola et al., 2019). The interaction occurs only with the GTP-bound form and it requires the presence of the prenyl moiety but it is negatively regulated by palmitoylation. It is currently unknown which are the enzymes responsible for K-Ras4A de/acylation. However, it has been suggested that palmitoylation may be in charge of a PM-resident enzyme, whereas mitochondrial depalmitoylation could be performed by APT1 or ABHD10 (Cao et al., 2019). In addition, a third lipid modification could act as an additional regulatory mechanism for K-Ras4A. Hence, Lin et al. have shown that K-Ras4A can also be reversibly acylated with palmitic acid at lysine residues located at the HVR (K182/184/185). Lysine acylation occurs on fully lipidated proteins and lipid removal, that promotes its transforming activity, is mediated by Sirtuin 2 (Jing et al., 2017) and inhibited by JH-T4 (Spiegelman et al., 2019) (Table 1). Lysine acylation has been also detected on H-Ras (K170) and N-Ras, but these proteins are not substrates of Sirtuin 2. Further work is required to elucidate the role lysine acylation on H-/N-Ras and to identify the enzymes that control this process.

## Membrane Microdomain Localization of Ras Proteins

Lipidation not only regulates the subcellular localization of Ras proteins, but also its lateral segregation and distribution between membrane microdomains, which is crucial for efficient signal transmission. Thus, the lateral heterogenous composition of cellular membranes results in the transient formation of distinct subcompartments: packed domains enriched with cholesterol and sphingolipids referred to as lipid ordered (l_o_) domains o rafts, and more fluid domains termed liquid disordered (l_d_) domains or non-rafts. In this second part, we will give a brief overview on Ras segregation in membrane subdomains (for a more detailed description see Erwin et al., 2017 and references herein).

Initial studies by Hancock et al. showed that **H-Ras** segregation within membrane microdomains was GDP/GTP-dependent. H-Ras resides in lipid rafts in its inactive form, but the active GTP-bound form as well as the active mutant HRasV12 migrate to l_d_ membranes (Prior et al., 2001; Rotblat et al., 2004), where they can activate proliferation and differentiation (Herrero et al., 2016; Figure 1B). However, GDP/GTP-dependent H-Ras partitioning may also be cell-specific (Agudo-Ibáñez et al., 2015). These different lateral segregations might regulate the biological output of H-Ras by a yet unknown mechanism. Thus, Ras signaling from rafts results in phosphorylation of epidermal growth factor receptor and cytosolic phospholipase A_2_, whereas signaling from fluid domains causes activation of kinase suppressor of Ras 1 (Casar et al., 2009). Moreover, H/N-Ras signals from lipid rafts or ER yield big tumors but with a reduced propensity to disseminate, whereas signaling from Golgi and disordered regions displayed higher migration rates (Agudo-Ibáñez et al., 2015; García-Ibáñez et al., 2020).

The effect of lipidation in membrane partitioning and clustering behavior of **N-Ras** has also been widely characterized. Initial studies in homogeneous membranes showed the formation of dimers (Güldenhaupt et al., 2012), that could be an initial step to the formation of small nanoclusters (Erwin et al., 2016). More complex systems gave, however, controversial results. Thus, the first insight into N-Ras microlocalization suggested that N-Ras was mainly found in rafts (Matallanas et al., 2003) at least in its GTP-form (Prior et al., 2001; Roy et al., 2005b).

A major advancement in the field came with the development of semisynthetic methods to obtain fully lipidated Ras proteins in quantities enabling biophysical studies (Triola et al., 2012). Hence, atomic force microscopy studies performed in heterogeneous model membranes revealed that N-Ras partitioning occurs preferentially into l_d_ domains followed by time-dependent clustering in domain (l_o_/l_d_) boundaries regions (Weise et al., 2009, 2010). No significant GDP/GTP-dependent partitioning was observed, although the inactive form showed stronger membrane association (Gohlke et al., 2010). Moreover, upon membrane binding, N-Ras showed free rotation of the G-domain, what may facilitate its interaction with effectors (Werkmuller et al., 2013). Accumulation in l_o_/l_d_ phase boundaries could also be observed in viral membrane extracts (Vogel et al., 2009) or using a FRET-based study (Shishina et al., 2018).

Recent breakthroughs have developed more sensitives techniques that revealed that N-Ras S-acylation is not restricted to the saturated palmitate but also includes the unsaturated palmitoleate. Characterization of palmitoleated N-Ras distribution in model membranes indicated a different behavior compared to the saturated N-Ras, showing a rapid membrane insertion and preferentially clustering in the l_d_ domains. Interestingly, these results suggest that S-acylation with different fatty acids may be an additional regulation point in N-Ras signaling (Schulte-Zweckel et al., 2019). The existence of thioesterases or PATs specifically committed to palmitoleated N-Ras remains elusive (Schulte-Zweckel et al., 2019). However, the fact that the 23 DHHCs have shown marked differences in fatty acid selectivity might suggest some substrate specificity (Greaves et al., 2017).

Currently, there is no information about the lateral segregation behavior of K-Ras4A. On the contrary, the splice variant K-Ras4B is better characterized. Thus, the polybasic **K-Ras 4B** preferentially distributes in l_d_ domains and spontaneous assembles to form new domains containing proteins and lipids (Weise et al., 2011). The presence of the prenyl group combined with the precise amino acid sequence of the polybasic region define the lipid composition of these nanoclusters (Zhou et al., 2017). The enrichments was independent of GDP/GTP loading but the active form showed bigger clusters (Kapoor et al., 2012). K-Ras4B distribution on the l_d_ domain was also observed in GUVs made from the envelope membrane of viral lipids (Weise et al., 2011) and protein-containing GPMVs (Erwin et al., 2016). Transport to the membrane is mediated by PDE6δ, whereas phosphorylation at Ser181 facilitates the PM dissociation (Zhang et al., 2017). Extraction from negatively charged membranes can also be performed in a GDP/GTP-independent manner by Calmodulin (Sperlich et al., 2016).

## Conclusions and Perspectives

It is becoming clear that the differential spatiotemporal distribution on organelles and subdomains has a key role in regulating the functional versatility of Ras proteins. As reversible lipidation is critical for maintaining the correct localization of H-/N- and probably KRas4A, a better understanding of the mechanism and dynamics by which S-acylation is controlled will provide new insights into the functional role of these modifications. Major outstanding questions still remain unanswered, such as how is the dynamic of lipid turnover regulated, how the presence of saturated or unsaturated fatty acids may influence protein function, which are all the enzymes involved in de/acylation and their selectivity profile or whether changes in the S-acylation (turnover rate, fatty acid identity) are linked with specific disease states. Furthermore, an increase in our knowledge of the mechanism and outcomes of protein S-acylation could lead to the identification of novel therapeutic opportunities.

## Author Contributions

CB-H wrote the manuscript. GT wrote and revised the manuscript. Both authors contributed to the article and approved the submitted version.

## Conflict of Interest

The authors declare that the research was conducted in the absence of any commercial or financial relationships that could be construed as a potential conflict of interest.
